# Supplementary material for: Predictors of Outcome after (Chemo)Radiotherapy for Node-Positive Oropharyngeal Cancer: The Role of Functional MRI
Source: Cancers (Basel). 2022 May 18;14(10):2477. doi: 10.3390/cancers14102477 (PMC9139324; doi:10.3390/cancers14102477)
Supplement: Supplementary file 1 [file cancers-14-02477-s001.zip › cancers-1696584-supplementary.pdf]

Supplementary Material: Predictors of Outcome after (Chemo)Radiotherapy for Node-Positive Oropharyngeal Cancer: The Role of Functional MRI

Pasqualina D'Urso *, Alessia Farneti, Laura Marucci, Simona Marzi, Francesca Piludu, Antonello Vidiri and Giuseppe Sanguineti

Materials and Method

Model building

Before building the models, the z-score normalization method was applied to standardize the (numerical) predictors: each variable was normalized as z=(x-<x>)/std, where x, <x>, and std were the variable value, mean value, and standard deviation, respectively. In this way, the variables were measured on the same scale and this allowed an improvement of ML algorithms efficiency. Different machine learning algorithms were trained on our dataset to find the most appropriate one. The families of ML algorithms used were: Decision Tree, Linear Discriminant, Logistic Regression, Naives Bayes, Support Vector Machine, K-Nearest Neighbor Classifiers and Ensamble Classifiers, as illustrated in Supplementary Figure 2.

An iterative optimization process was used to select the most appropriate hyperparameters of the Decision Tree classifier (i.e. tree depth and split criterion technique), which was found as the best ML algorithm on our dataset. Specifically, the maximum number of split to control the depth of the tree was 4 and the split criterion was the maximum deviance reduction.

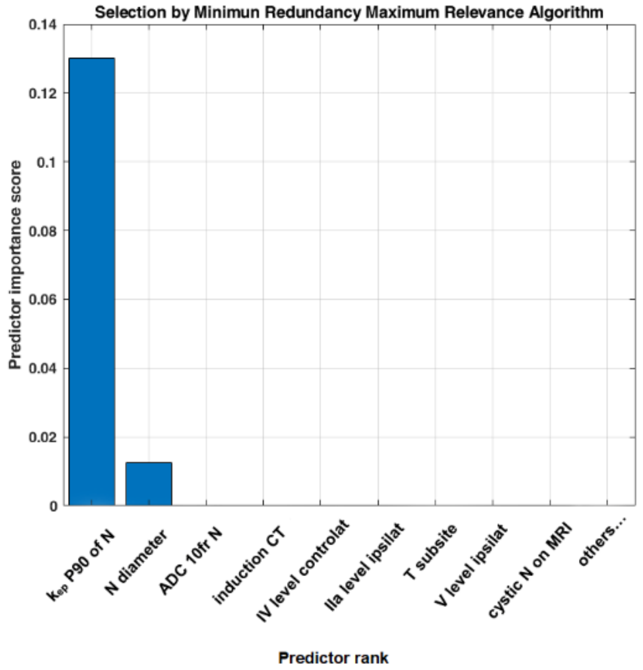

Figure S1. Bar plot of predictor importance scores after selection by Minimum Redundancy Maximum Relevance (MRMR) algortithm**.**

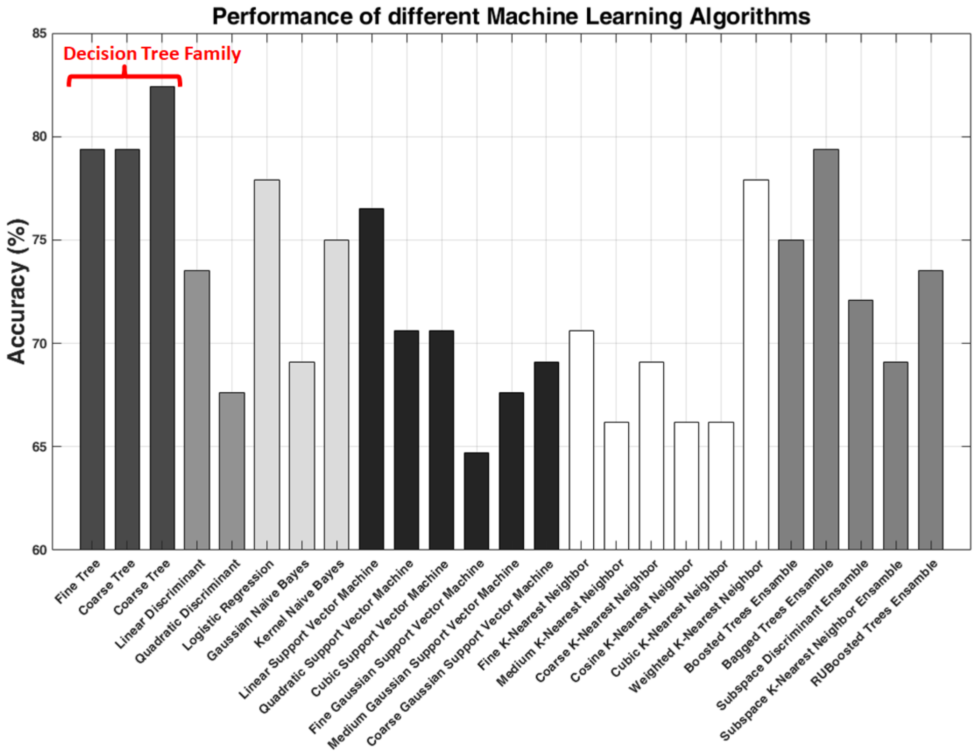

Figure S2. Bar plot of the accuracies obtained by different families of ML algorithms.

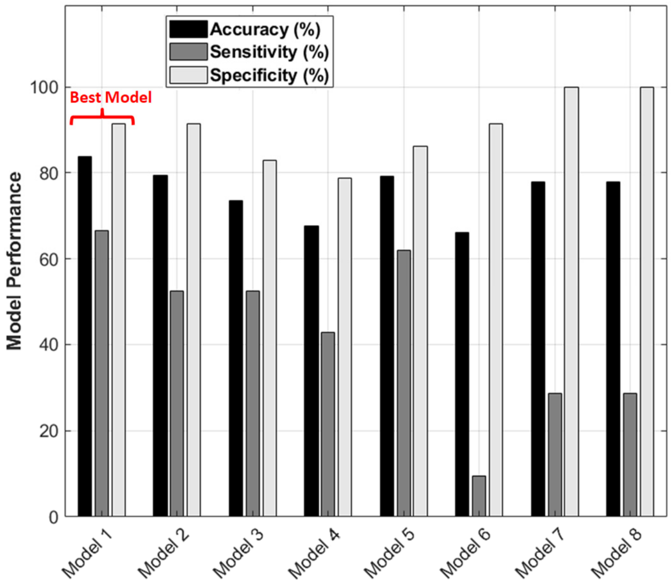

Figure S3. Predictive performances of various models obtained from different combination of the most relevant predictors. Predictor names of each model are described in Supplementary Table S1.

Table S1. Predictors included in the models shown in Figure S3.

	Predictors
**Model 1 (Best Model)**	*N Diameter, N K_{ep} P90*
**Model 2**	*Smoke, N Diameter, Cystic N on MR, ENE, ADC_N(%), p16 status*
**Model 3**	*Smoke, N Diameter, Cystic N on MR, ENE p16 status*
**Model 4**	*Smoke, N Diameter, Cystic N on MR, ENE*
**Model 5**	*N Diameter, Cystic N on MR, ENE*
**Model 6**	*Smoke, N Diameter, ENE*
**Model 7**	*Smoke, N Diameter, ENE, p16 status*

**Model 8**	*Smoke, N Diameter, p16 status*

Table S2. Comparison between predictions of the models shown in Figure S3.

Model	1	2	3	4	5	6	7	8
**1**	1.000	0.791	0.804	0.167	0.804	**0.021**	0.210	0.210
**2**	0.791	1.000	0.581	0.057	0.629	**0.007**	0.065	0.065
**3**	0.804	0.581	1.000	**0.031**	1.000	0.108	0.383	0.383
**4**	0.167	0.057	**0.031**	1.000	0.210	0.523	0.832	0.832
**5**	0.804	0.629	1.000	0.210	1.000	0.108	0.359	0.359
**6**	**0.021**	**0.007**	0.108	0.523	0.108	1.000	0.125	0.125
**7**	0.210	0.065	0.383	0.832	0.359	0.125	1.000	1.000
**8**	0.210	0.065	0.383	0.832	0.359	0.125	1.000	1.000

P-values refer to the mid-*p*-value McNemar test. Statistically significant *p*-values are **bold.**

Filename:	cancers-1696584 supplementary material updated.docx
Directory:	E:\5.17\computation-1719131
Template:	C:\Users\MDPI\AppData\Roaming\Microsoft\Templates\Normal.dotm
Title:	S
Subject:	
Author:	MDPI
Keywords:	
Comments:	
Creation Date:	4/11/2022 2:31:00 PM
Change Number:	6
Last Saved On:	5/18/2022 3:00:00 PM
Last Saved By:	MDPI
Total Editing Time:	44 Minutes
Last Printed On:	5/18/2022 4:24:00 PM

As of Last Complete Printing

Number of Pages: 3

Number of Words: 465 (approx.)

Number of Characters: 2,483 (approx.)
